# Association Between Gestational Diabetes Mellitus and Future Risk of Kidney Stones

**DOI:** 10.3389/fpubh.2022.843383

**Published:** 2022-02-14

**Authors:** Yuanyuan Mao, Wenbin Hu, Li Liu, Qin Liu

**Affiliations:** ^1^Medical College of Soochow University, Suzhou, China; ^2^Department of Obstetrics and Gynecology, The First People's Hospital of Kunshan Affiliated With Jiangsu University, Suzhou, China; ^3^Department of Chronic and Noncommunicable Disease Control and Preventions, The Kunshan Center for Disease Control and Prevention, Suzhou, China

**Keywords:** kidney stones, National Health and Nutrition Examination Survey, gestational diabetes mellitus, type 2 diabetes, hypertension, metabolic syndrome

## Abstract

**Objective:**

The association between history of gestational diabetes mellitus (GDM) and risk of kidney stones has not been reported. GDM increases the risk of long-term complications including diabetes, hypertension and metabolic syndrome, which are risk factors of kidney stones. This study aimed to explore the association between previous GDM and odds of kidney stones.

**Methods:**

Women (age ≥ 20 years) who had delivered at least one live birth were included from the 2007–2018 National Health and Nutrition Examination Survey cohort (*N* = 12,003). Patients with kidney stones and history of GDM were identified by in-home interview for all participants. Subgroup analyses were conducted by age, race/ethnicity, postpartum duration and status of hypertension, obesity, current diabetes and metabolic syndrome.

**Results:**

Previous GDM was positively associated with odds of kidney stones [multivariate-adjusted odds ratio (95% confidence interval): 1.41 (1.13–1.77)], and the association was stronger with odds of passing 2 or more times of kidney stones [1.72 (1.31–2.26)]. In subgroup analyses, the association between previous GDM and odds of kidney stones was significant in women within 15 years of a pregnancy complicated by GDM [1.54 (1.12–2.11)], in obese participants [1.56 (1.18–2.06)], in women without hypertension [1.49 (1.07–2.08)], current diabetes [1.38 (1.02–1.87)] and metabolic syndrome [1.56 (1.10–2.19)], in women of Non-Hispanic White [1.59 (1.15–2.18)] and in women aged more than 50 year [1.45 (1.02–2.07)].

**Conclusions:**

Previous GDM was positively associated with odds of kidney stones, and the association was independent of type 2 diabetes, hypertension and metabolic syndrome.

## Introduction

Gestational diabetes mellitus (GDM) is currently the most common medical complication of pregnancy (1). Globally, the prevalence of GDM is 14.7% according to the International Association of Diabetes and Pregnancy Study Groups criteria (2), and the prevalence could vary substantially depending on population characteristics such as age, race/ethnicity, obesity, and type 2 diabetes mellitus prevalence in the background population (2). Kidney stones are the third most common urological disease with a prevalence of about 15% worldwide (3), and the prevalence and incidence of kidney stones is increasing in the United States and other parts of the world (4). In addition, the estimated 5-year recurrence rate is up to 50% (5). Patients with kidney stones have twice the risk of chronic kidney disease or end stage renal disease, and the risk is higher for females (6). The costs associated with stone disease have increased from $2 billion to over $10 billion from 2000 to 2006 in the United States alone (7).

GDM increases the risk of long-term complications including diabetes (8, 9), cardiovascular diseases (10, 11), metabolic syndromes (12) and cancer (13). However, the association between previous GDM and risk of kidney stones has not been reported. Inflammation and oxidant-antioxidant imbalance may play crucial roles in the development of kidney stones (3). Metabolic syndrome, diabetes, obesity and hypertension are established risk factors for kidney stone formation (7), and maternal obesity, type 2 diabetes, metabolic syndrome are also major risk factors for GDM development (1), supporting the potential link between GDM and development of kidney stones. A meta-analysis of prospective cohort studies showed that the summary relative risk was 1.16 (95% CI 1.03–1.31, *I*^2^ = 51%, *n* = 10) for participants with type 2 diabetes compared to participants without type 2 diabetes (14). Based on the above-mentioned findings, we hypothesized that previous GDM could be positively associated with odds of kidney stones. In addition, given the prevalence of both GDM and kidney stones varies much depending on population characteristics such as age and race/ethnicity (2, 15, 16), and the effect of GDM on long-term complications maybe differential by years after pregnancy (8), we conducted stratified analyses to explore the possible interactions between GDM and these stratified factors on kidney stones. In addition, because GDM increases the risk of long-term complications including diabetes, hypertension and metabolic syndrome which are risk factors of kidney stones (7), we also conducted stratified analyses by the presence of these chronic diseases to explore whether these chronic diseases could account for the association between previous GDM and odds of kidney stones.

## Materials and Methods

### Study Populations

As a major program of the National Center for Health Statistics, the National Health and Nutrition Examination Survey (NHANES) cohort is designed to assess the health and nutritional status of a nationally representative sample of about 5,000 persons in each 2-year cycle. We used data from six cycles of the NHANES cohort (2007/2008 to 2017/2018), as these cycles specifically provided information of GDM. All women aged 20 years or older and with at least one live birth were potentially eligible for this analysis. Women who did not provide information of GDM and kidney stones, who were diagnosed with diabetes prior to a diagnosis of GDM, and women having kidney stones at the time of pregnancy complicated by GDM were excluded from this analysis.

### GDM and Kidney Stones

Women who had GDM during pregnancy were identified if they answered yes to the following question: “During your pregnancy, were you ever told by a doctor or other health professional that you had diabetes, sugar diabetes or gestational diabetes?” (17). Patients with kidney stones were identified with the questions of “Have you ever had kidney stones?”, and “How many times have you passed a kidney stone?” We considered any subject who reported a history of stone disease including symptomatic stone disease (16).

### Covariates

According to the previous studies (14), the following covariates were included: age (in 10-year increments), race/ethnicity (Mexican–American, Other Hispanic, Non-Hispanic White, Non-Hispanic Black, Other Races), annual family income (< $20,000, $20,000–$44,999, $45,000–$74,999, ≥$75,000), education ( ≤ high school, Some college or AA degree, ≥College graduate), body mass index (under/normal weight: <25 kg/m^2^, overweight: 25 to <30 kg/m^2^, obesity: ≥30 kg/m^2^), hypertension, current diabetes, physical activity (vigorous/ moderate recreational activities for at least 10 min continuously per week), smoking (current smoker, former smoker, never smoker), uric acid and daily intake of total energy, total water drank, calcium, phosphate, sodium, alcohol and vitamin C. Current diabetes was defined using a self-reported diagnosis of diabetes outside pregnancy or, if diabetes was not previously diagnosed, by a hemoglobin A_1c_ level ≥ 6.5%, a fasting plasma glucose level ≥ 126 mg/dL, or 2-h plasma glucose ≥ 200 mg/dL, or taking diabetic pills to lower blood sugar (18). According to the 2017 American College of Cardiology/American Heart Association Task Force on Clinical Practice Guidelines, hypertension was defined if they were taking antihypertensive medication, if their systolic blood pressure exceeded 130 mmHg, or if their mean diastolic blood pressure exceeded 80 mmHg (mean values of three measurements) (19).

In addition, as both GDM and kidney stones are associated with metabolic syndromes (1, 7, 12), we conducted a sensitivity analysis in which we adjusted for metabolic syndrome rather than hypertension, obesity and diabetes to determine whether metabolic syndrome could account for the association. Any 3 of the 5 following metabolic-related disorders constitute diagnosis of metabolic syndrome (20): elevated waist circumference (≥102 cm in men, ≥88 cm in women), elevated triglycerides (≥150 mg/dL), reduced HDL-C (<40 mg/dL in men, <50 mg/dL in women), elevated blood pressure (≥130 mm Hg systolic blood pressure, ≥85 mm Hg diastolic blood pressure) and elevated fasting glucose (≥100 mg/dL).

### Statistical Analysis

Weighted logistic regression was used to calculate the odds ratios (95% confidence interval) [OR (95% CI)] of kidney stones for women with previous GDM compared with the control groups. We calculated three different logistic regression models. Model 1 was adjusted for age, race/ethnicity and body mass index. Model 2 included the covariates of model 1 with additional adjustment for education, family income, hypertension and current diabetes. Model 3 included the covariates of model 2 with additional adjustment for alcohol drinking, smoking, physical activity, uric acid and dietary intakes of energy, total water, calcium, phosphate, sodium, potassium and vitamin C. New multi-year sample weight was computed by dividing the 2-year sample weights by 6. Stratified analyses were conducted by age ( ≤ 50, >50 years), race/ethnicities (Non-Hispanic White, others), postpartum duration ( ≤ 15 years, >years), hypertension (yes, no), obesity (yes, no) and current diabetes (yes, no). We also conducted a sensitivity analysis in which we adjusted for metabolic syndrome rather than hypertension, obesity and diabetes to determine whether metabolic syndrome could account for the association. Tests for interactions were performed by using cross-product terms of GDM with these stratified factors. All analyses were conducted with Stata 12.0, and *P* ≤ 0.05 was considered statistically significant.

## Results

A total of 17,907 women aged 20 years or older were included in the 2007/2008 to 2017/2018 NHANES. After excluding those who did not have at least one live birth (*N* = 5,692), who were diagnosed with diabetes prior to GDM (*N* = 48), who did not answer to the question regarding history of GDM or answered “borderline” (*N* = 139), who did not response to the question of ever having kidney stones (*N* = 24), and who had kidney stones at the time of pregnancy complicated by GDM (*N* = 1), 12,003 women were finally included in this analysis. The weighted prevalence of GDM and kidney stones was 7.99 and 9.91%, respectively. Women who had GDM during pregnancy tend to be younger, had higher family income and education level, and showed higher prevalence of obesity, current diabetes and kidney stones, but tend to have lower prevalence of hypertension. For the 2,412 patients with current diabetes, 48 subjects were identified to have type 1 diabetes (currently using insulin and diagnosed with diabetes under age 30) (21). The race/ethnicity also differs significantly between women who had GDM during pregnancy and the controls (Table 1).

**Table 1:** Characteristics of the 2007–2018 NHANES adults by history of gestational diabetes mellitus (GDM).

	**Patients with GDM (926)**	**Controls (11,077)**	***P* ^a^**
Age [years, mean (SD)]	45.47 (12.30)	53.48 (16.76)	<0.01
Race/Hispanic origin (%)			<0.01
Mexican American	21.92	15.78	
Other Hispanic	11.34	11.67	
Non-Hispanic White	35.75	40.76	
Non-Hispanic Black	17.71	22.13	
Other Race	13.28	9.66	
Annual family income (%)			<0.01
< $20,000	20.56	26.56	
$20,000–$34,999	33.45	33.74	
$35,000–$74,999	18.87	17.58	
≥$75,000	27.12	22.09	
Education (%)			<0.01
≤ High school	43.63	50.12	
Some college or AA degree	35.96	30.65	
≥College graduate	20.41	19.23	
Vigorous/moderate recreational activities for at least 10 min continuously per week (%)	14.15	13.68	0.89
Smoking			0.36
Current smoker	18.79	17.47	
Former smoker	18.03	19.66	
Never smoker	63.17	62.87	
Obesity (%)	56.07	43.06	<0.01
Hypertension (%)	48.16	56.22	<0.01
Current diabetes (%)	36.39	18.69	<0.01
Kidney stones (%)	12.96	8.81	<0.01
Metabolic syndrome (%)	45.66	37.44	<0.01
Uric acid (mg/dL)	4.94	4.93	0.79
Daily intake [M (SD)]			
Total energy (kcal)	1,883.04 (873.88)	1,757.65 (748.04)	<0.01
Total water drank (g)	1,232.69 (1,187.74)	1,039.10 (1,055.41)	<0.01
Calcium (mg)	876.67 (522.43)	810.84 (474.48)	<0.01
Phosphate (mg)	1,233.16 (590.92)	1,131.01 (515.95)	<0.01
Sodium (mg)	3,131.18 (1,576.97)	2,879.43 (1,428.66)	<0.01
Alcohol (g)	5.19 (19.10)	4.84 (16.34)	0.54
Vitamin C (mg)	73.81 (84.47)	75.55 (80.56)	0.54

*M, Mean values; SD, standard deviation*.

a *t-test was performed for continuous variables, and Chi-square test was performed for categorical variables*.

The findings between previous GDM and kidney stones were similar across the three statistical models, while the magnitude of the observed association was attenuated slightly in model 2 and model 3. In model 3, previous GDM was associated with higher odds of kidney stones [OR (95% CI): 1.41 (1.13–1.77), *P* < 0.01], and the association was stronger with odds of passing 2 or more times of kidney stones [1.72 (1.31–2.26), *P* < 0.01]. In subgroup analyses, the positive association between previous GDM and kidney stones was also evident in women within 15 years of a pregnancy complicated by GDM [1.54 (1.12–2.11), *P* < 0.01], in women without hypertension [1.49 (1.07–2.08), *P* < 0.05], in obese women [1.56 (1.18–2.06), *P* < 0.01], in women without current diabetes [1.38 (1.02–1.87), *P* < 0.05], in women of Non-Hispanic White [1.59 (1.15–2.18), *P* < 0.01], and in women of age > 50 years [1.45 (1.02–2.07), *P* < 0.05]. However, the interactions between previous GDM and the above-mentioned stratified factors were not significant (all *P*_forinteraction_ > 0.05) (Table 2).

**Table 2:** Odds ratios (95% confidence intervals) of kidney stones associated with previous gestational diabetes mellitus.

**Groups**	**Odds ratios (95% confidence intervals)**					** ${\text{P}}_{\text{forinteraction}}^{\text{a}}$ **
	**Cases with kidney stones/*N***	**Model 1**	**Model 2**	**Model 3**	**Sensitivity analysis**	
Overall	1,096/12,003	1.50 (1.22–1.85)^**^	1.39 (1.11–1.72)^**^	1.41 (1.13–1.77)^**^	1.57 (1.26–1.96)^**^	
Postpartum duration						0.53
<15 years	1,032/11,529	1.69 (1.25–2.30)^**^	1.63 (1.20–2.23)^**^	1.54 (1.12–2.11)^**^	1.65 (1.21–2.26)^**^	
≥15 years	1,040/11,551	1.36 (1.03–1.80)^*^	1.22 (0.92–1.64)	1.32 (0.98–1.79)	1.50 (1.12–2.02)^**^	
Hypertension						0.79
Yes	697/6,673	1.42 (1.07–1.88)^*^	1.28 (0.95–1.73)	1.33 (0.98–1.82)	1.49 (1.11–2.02)^**^	
No	399/5,330	1.60 (1.17–2.18)^**^	1.52 (1.10–2.09)^*^	1.49 (1.07–2.08)^*^	1.67 (1.20–2.31)^**^	
Obesity						0.62
Yes	516/6,767	1.62 (1.25–2.10)^**^	1.51 (1.15–1.98)^**^	1.56 (1.18–2.06)^**^	1.70 (1.29–2.23)^**^	
No	580/5,236	1.29 (0.90–1.85)	1.18 (0.81–1.73)	1.17 (0.78–1.74)	1.27 (0.86–1.88)	
Current diabetes						0.96
Yes	309/2,407	1.27 (0.90–1.78)	1.28 (0.91–1.81)	1.30 (0.91–1.86)	1.34 (0.94–1.92)	
No	787/9,596	1.40 (1.06–1.85)^*^	1.42 (1.07–1.90)^*^	1.38 (1.02–1.87)^*^	1.40 (1.04–1.89)^*^	
Race/ethnicities						0.81
Non-Hispanic White	549/4,846	1.57 (1.16–2.13)^**^	1.51 (1.10–2.06)^*^	1.59 (1.15–2.18)^**^	1.66 (1.21–2.27)^**^	
Others	547/7,157	1.44 (1.08–1.93)^*^	1.28 (0.95–1.74)	1.24 (0.90–1.72)	1.47 (1.07–2.01)^*^	
Age, years						0.90
≤ 50	432/5,458	1.61 (1.22–2.11)^**^	1.40 (1.05–1.86)^*^	1.33 (0.99–1.79)	1.50 (1.12–2.00)^**^	
>50	664/6,545	1.46 (1.05–2.03)^*^	1.41 (1.01–1.99)^*^	1.45 (1.02–2.07)^*^	1.60 (1.12–2.26)^**^	
Metabolic syndrome^b^						0.93
Yes	546/4,908	1.58 (1.19–2.08)^**^	1.60 (1.21–2.13)^**^	1.57 (1.18–2.10)^**^	—	
No	550/7,095	1.46 (1.06–2.01)^*^	1.50 (1.09–2.08)^*^	1.56 (1.10–2.19)^*^	—	

* *P < 0.05*.

** *P < 0.01*.

*Model 1: adjusted for age, race/ethnicity and body mass index*.

*Model 2: adjusted for covariates in model 1 and education, family income, hypertension and current diabetes*.

*Model 3: adjusted for covariates in model 2 and alcohol drinking, smoking, physical activity, uric acid and dietary intakes of energy, total fluid, calcium, phosphate, sodium, potassium and vitamin C*.

*Sensitivity analysis: adjusted for age, race/ethnicity, education, family income, alcohol drinking, smoking, physical activity, uric acid and dietary intakes of energy, total fluid, calcium, phosphate, sodium, potassium and vitamin C, and metabolic syndrome*.

a *P-values for interaction analyses in model 3*.

b *In subgroup analysis by metabolic syndrome, body mass index, hypertension and current diabetes were not included in the above models*.

In sensitivity analysis in which we adjusted for metabolic syndrome rather than hypertension, obesity and diabetes, previous GDM was also associated with higher odds of kidney stones [1.57 (1.26–1.96), *P* < 0.01] in model 3. In addition, the association was evident in both women who had metabolic syndrome [1.57 (1.18–2.10), *P* < 0.01] and who did not have metabolic syndrome [1.56 (1.10–2.19), *P* < 0.05] (*P*_forinteraction_ = 0.93) (Table 2). In addition, 64 participants were defined as patients with current diabetes because they were taking diabetic pills to lower blood sugar. Some of lowering sugar pills is used not only in diabetic people, but also obese non-diabetic women. However, the results remain unchanged when these 64 participants were included in the group of non-diabetic women in model 3 [1.41 (1.12–1.77), *P* < 0.01].

## Discussion

To our knowledge, this is the first study to explore the association between history of GDM and kidney stones. After adjusting for other covariates, results from the national survey cohort suggested that previous GDM was independently associated with higher odds of kidney stones, and the association was independent of type 2 diabetes, hypertension and metabolic syndrome. Some differences in the association between previous GDM and kidney stones were found in stratified analyses by key population characteristics that are associated with the prevalence of GDM and kidney stones; however, these differences were not significant.

Several potential mechanisms could explain an association between history of GDM and kidney stones. GDM increases the risk of long-term complications including diabetes (8, 9). In particular, women who had GDM during pregnancy have a 7–10-fold increased risk of developing type 2 diabetes and the percentage diagnosed with type 2 diabetes was 12% higher for each additional year after pregnancy (8, 9, 22). A meta-analysis of 10 prospective cohort studies showed a 16% increase in the relative risk of kidney stones among diabetes patients compared to persons without diabetes (14). In our study, a weaker but significant association between previous GDM and kidney stones was also found after adjusting for other covariates including current diabetes. Furthermore, the magnitude of association between previous GDM and kidney stones was larger in women without current diabetes than those with current diabetes. These findings suggested that current diabetes cannot fully account for the observed association. Results from our study are consistent with those from a previous follow-up study indicating that the risk of cardiovascular disease associated with GDM was not fully dependent upon the development of type 2 diabetes (23). In addition, a retrospective cohort study showed that a history of nephrolithiasis was associated with higher risks of GDM [OR (95% CI): 3.1 (1.8–5.3)] and preeclampsia [2.2 (1.3–3.6)], suggesting that stone formation is a marker of metabolic diseases and supporting the link between GDM and kidney stones (24).

Second, in addition to diabetes, obesity, hypertension and metabolic syndrome are also risk factors for stone formation (7). Results from a recent review suggested that women with previous GDM have significantly higher blood pressure, body mass index, total cholesterol, LDL cholesterol, triglycerides, glucose and significantly lower HDL cholesterol (10). In this analysis, women with previous GDM also had significantly higher prevalence of obesity, diabetes and metabolic syndrome, while had significantly lower prevalence of hypertension. These findings suggested that the long-term effects of GDM on other components of metabolic syndrome might be much more evident that on hypertension. However, the association between previous GDM and kidney stones was stronger in sensitivity analysis adjusting for metabolic syndrome, and the association was also significant in women without metabolic syndrome. Therefore, metabolic syndrome cannot also not fully account for the observed association. As non-Hispanic White individuals, obese individuals and older subjects are much more likely to report a history of kidney stones (16), it is theoretically reasonable to find a stronger association in these population groups.

Third, kidney stones form on a foundation of calcium phosphate called Randall's plaques present on the renal papillary surface. The molecular aspect of nephrolithiasis development include inflammation, oxidant–antioxidant imbalance, angiogenesis, purine metabolism and urea cycle disorders (3). The three central features of pregnancies complicated by GDM include insulin resistance, low-grade inflammation and endothelial cell dysfunction (25). In the Diabetes and Women's Health study, women with a GDM history had significantly higher estimated glomerular filtration rate and urinary albumin-to-creatinine ratio 9–16 years postpartum, indicating early stages of glomerular hyperfiltration and renal damage (26). Women who developed type 2 diabetes after a pregnancy complicated by GDM also had an increased risk renal dialysis [hazard ratio (95% CI): 7.52 (5.24–10.81)] (23). In addition, GDM was also found as a significant risk factor for future maternal renal morbidity in a study with a mean follow-up duration of 11.2 years (27). GDM alone in the absence of subsequent diabetes was associated with microalbuminuria in the Kidney Early Evaluation Program (28). These findings suggested that GDM could be a risk factor for renal damage.

There are several limitations. First, we were unable to determine the causality in this cross-sectional study. However, the prevalence of kidney stones increases with age (16). In this study, the mean age of patients told to have GDM was 28.40 years (SD: 6.58), and the mean age of participants at the time of survey was 52.87 years (SD: 16.60). In addition, women having kidney stones at the time of pregnancy complicated by GDM were also excluded from this analysis. Second, stones composed of calcium oxalate mixed with calcium phosphate, struvite, uric acid and cystine account for ~80, 10, 9, and 1% of stones (7), respectively. Therefore, it is necessary to determine if previous GDM is associated with the risk of certain stone types but not others in further studies. Third, history of GDM and kidney stones were self-reported, and previous medical records about GDM are not available in the NHANES. However, these data from NHANES are considered to be valid and have been widely used in epidemiological studies (16, 17, 29, 30), and misclassification of patients with undiagnosed GDM and kidney stones as controls would have biased the study results toward the null.

## Conclusion

In conclusion, findings from this nationally representative cohort suggested that previous GDM was positively associated with odds of kidney stones, and the association was independent of type 2 diabetes, hypertension and metabolic syndrome. These findings deserve to be confirmed by prospective cohort studies.

## Data Availability Statement

The datasets generated during and/or analyzed during the current study are available in the NHANES: https://www.cdc.gov/nchs/nhanes/.

## Ethics Statement

The studies involving human participants were reviewed and approved by National Center for Health Statistics Research Ethics Review Board. The patients/participants provided their written informed consent to participate in this study.

## Author Contributions

YM and QL designed the study. WH conducted the statistical analysis. YM, LL, and QL drafted the manuscript. QL made critical revisions. All authors have approved the final article.

## Funding

This work was supported by grants from the Maternal and Child Health Research Project of Jiangsu Province (No. F201720) and the Development Science and Technology Project of Kunshan (No. KS1646).

## Conflict of Interest

The authors declare that the research was conducted in the absence of any commercial or financial relationships that could be construed as a potential conflict of interest.

## Publisher's Note

All claims expressed in this article are solely those of the authors and do not necessarily represent those of their affiliated organizations, or those of the publisher, the editors and the reviewers. Any product that may be evaluated in this article, or claim that may be made by its manufacturer, is not guaranteed or endorsed by the publisher.
